# Representations in vision and language converge in a shared, multidimensional space of perceived similarities

**DOI:** 10.1167/jov.26.5.7

**Published:** 2026-05-20

**Authors:** Katerina M. Simkova, Adrien Doerig, Clayton Hickey, Ian Charest

**Affiliations:** 1 Centre for Human Brain Health and School of Psychology, University of Birmingham, Birmingham, UK; 2 Department of Education and Psychology, Freie Universität Berlin, Berlin, Germany,; 3 CerebrUM, Département de Psychologie, Université de Montréal, Montréal, Québec, Canada; 4 Bernstein Center for Computational Neuroscience, Berlin, Germany

**Keywords:** similarity judgments, conceptual representation, scene perception, language, large language models, AlexNet, representational similarity

## Abstract

Humans can effortlessly describe what they see, yet establishing a shared representational format between vision and language remains a significant challenge. Emerging evidence suggests that human brain representations in both vision and language are well predicted by semantic feature spaces obtained from large language models (LLMs). This raises the possibility that sensory systems converge in their inherent ability to transform their inputs onto shared, embedding-like representational space. However, it remains unclear how such a space manifests in human behavior. To investigate this, 63 participants performed behavioral similarity judgments separately on 100 natural scene images and 100 corresponding sentence captions from the Natural Scenes Dataset. We found that visual and linguistic similarity judgments not only converge at the behavioral level but also predict a remarkably similar network of functional magnetic resonance imaging brain responses evoked by viewing the natural scene images. Furthermore, computational models trained to map images onto LLM-embeddings outperformed both category-trained and AlexNet controls in predicting the behavioral similarity structure. These findings demonstrate that human visual and linguistic similarity judgments are grounded in a shared, modality-agnostic representational structure that mirrors how the visual system encodes experience. The convergence between sensory and artificial systems observed here suggests a common capacity of how conceptual representations are formed—not as arbitrary products of first order, modality-specific input, but as structured representations that reflect the stable, relational properties of the external world.

## Introduction

Humans effortlessly translate between what they see and what they say. This intuition has motivated the hypothesis of a shared high-level representational space for vision and language—a common code that represents concepts independent of whether they enter through the eyes or words (Nikolaus, Mozafari, Asher, Reddy, & VanRullen, 2024; Tang, Du, Vo, Lal, & Huth, 2023). Computational research has established a universal structure that explains how disparate streams of information coalesce into unified cognition (Liu, Ma, Zhou, Zhu, & Zheng, 2023; Tian, Krishnan, & Isola, 2020). As artificial systems are exposed to increasing amounts of naturalistic data, they appear to converge in their ability to recognize the underlying statistical structure of diverse inputs (Huh, Cheung, Wang, & Isola, 2024; van Rossem & Saxe, 2024). However, it remains unclear whether a similar universal structure emerges in the human brain and behavior. Our sensory diets are complex and unconstrained, making direct comparison of internal representations difficult.

Recent advances suggest that embeddings from large language models (LLMs) may capture a common representational code for concepts, regardless of whether they are conveyed through vision or language. Evidence shows that LLM embeddings of natural scene captions align with brain responses elicited when individuals view corresponding images (Doerig et al., 2025; Lahner et al., 2024; Tang et al., 2023). Aligning visual and linguistic representations provides even better predictors of visual responses in the brain (Wang, Kay, Naselaris, Tarr, & Wehbe, 2023), and improves performance in multiple computational tasks (Lu, Batra, Parikh, & Lee, 2019; Pramanick et al., 2022; Radford et al., 2021). The success of LLMs may stem from their exposure to massive linguistic corpora, enabling them to internalize the statistical structure of world knowledge (Hernandez, Kaplan, Henighan, & McCandlish, 2021; Kaplan et al., 2020). Similarly, the human brain integrates visual and linguistic information to form a robust understanding of its environment (B. Liu et al., 2025; Q. Liu, van Paridon, & Lupyan, 2025). Both systems thus may converge toward a similar representation capturing the complexity of the world.

These developments are paralleled in language research, where growing evidence shows that LLMs are strong predictors of natural language representations in the brain (Caucheteux & King, 2022; Dong & Toneva, 2023; Toneva & Wehbe, 2019). Some evidence suggests that their feature space also aligns with predictive processes (Goldstein et al., 2021; Schrimpf et al., 2021; Tuckute, Kanwisher, & Fedorenko, 2024) and high-level comprehension of narratives (Caucheteux, Gramfort, & King, 2022; Russo et al., 2022). (Nikolaus et al., 2024) further showed that encoding models trained on visual brain activity generalize to activity during natural language comprehension and vice versa. This converging evidence suggests that LLM embeddings provide a promising model of a shared representational structure—one that compresses information from different modalities.

In this study, we collected similarity judgments for natural scene images and sentence captions describing the same scenes to explore whether a shared representational format emerges in behavior. While recent work has demonstrated alignment between similarity judgments of action videos and their descriptions (Dima, Janarthanan, Culham, & Mohsenzadeh, 2024) and shown that LLMs capture aspects of human semantic structure (Du et al., 2025), it remains unclear whether such cross-modal similarity structure reflects a common neural representational format. We address this question in several ways. First, we begin with a carefully sampled set of natural scenes, ensuring coverage of diverse conceptual domains. Using the multiple arrangement (MA) method, we map how these concepts are organized in context, revealing fine-grained structure within behavioral similarity spaces. We then use representational similarity analysis (Kriegeskorte, Mur, & Bandettini, 2008) to link these behavioral spaces to brain activity of an independent participant group who viewed the same images, asking whether linguistic similarity judgments from individuals who never saw the images can nonetheless predict the neural geometry of visual encoding. Finally, we evaluate which computational models best approximate human conceptual structure, contrasting modality-specific models with those projecting visual inputs into language-based embedding spaces. To anticipate, our study reveals a universal representational structure that links behavior, brain, and computational models into a common conceptual space.

## Methods

### Participants

Sixty-six participants (23 ± 4 years of age) with normal or corrected-to-normal vision were recruited for the experiment. Three participants were excluded from analysis as they did not complete all three experimental sessions resulting in 63 participants left for analysis. Participants provided informed consent as part of a protocol approved by the University of Birmingham Research Ethics Committee and were awarded £8/h for participation.

### Stimuli

To ensure that the stimulus set covered a broad range of high-level semantic structure, we obtained stimuli from the Special100 subset of the Natural Scenes Dataset (NSD; (Allen et al., 2022), which consists of 100 natural scene images and their corresponding sentence captions. The Special100 images were selected from the shared set of 515 NSD images using a greedy algorithm (Allen et al., 2022) that maximizes coverage of high-level semantic space, as defined by embeddings of the images’ sentence captions. The original captions were collected from five human annotators as part of the Microsoft Common Objects in Context database (Lin et al., 2014). For each image, we identified the most frequent words and phrases across annotators and combined them to generate a single representative sentence caption.

### Experimental design

**Figure 1.**
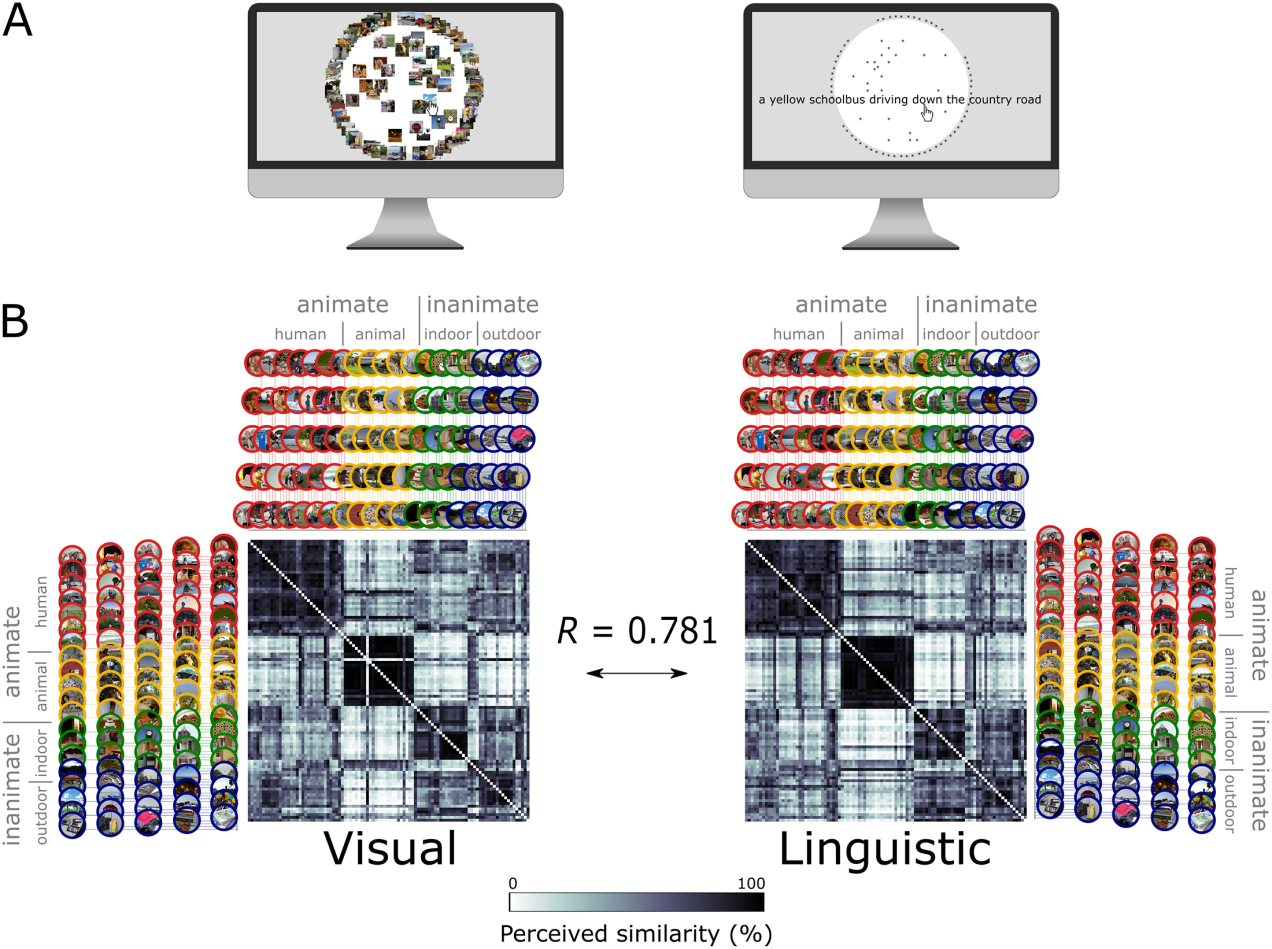
**Experimental design of the MA tasks.** (**A**) Participants completed the MA task either on 100 natural scene images (visual modality left) or 100 sentence captions describing the images (linguistic modality right). To handle the large set of sentence captions, we adapted the MA method such that every caption was depicted as an asterisk and only the item currently being dragged was displayed. (**B**) The rank transformed subject-averaged modality-specific RDMs across all sessions were correlated to investigate the representational overlap between the visual and linguistic similarity judgments. We averaged modality-specific RDMs across participants before calculating Spearman correlation of these results, which generated a correlation of 0.781, suggesting strong representational overlap. The icons are color-coded to differentiate between categories (red, human; yellow, animal; green, indoor; blue, outdoor).

To recover the representational geometry underlying visual and linguistic similarity judgments, participants completed the visual, linguistic, and control MA tasks in three in-person sessions scheduled at least 7 days apart (average time between sessions 8.3 ± 3.3 days). The experiment was hosted by the web-based platform Meadows (https://meadows-research.com/) and administered on a PC with a 25” monitor. The visual MA task (Figure 1A, left) followed the standard procedure as introduced in (Kriegeskorte & Mur, 2012). Briefly, 100 natural scene images were displayed around a white circular arena. Participants were then instructed to arrange the images inside the white arena according to their similarity using mouse drag-and-drop operations such that images perceived as more similar are placed closer together while images perceived as less similar are placed further apart. The linguistic MA task (Figure 1A, right) was conceptually similar, but involved the arrangement of sentence captions which allowed us to recover the representational geometry of the images’ linguistic referents. To reduce the clutter introduced by the display of numerous lengthy sentences around the MA arena, we adapted the MA method such that every sentence was initially displayed as an asterisk. The sentence appeared only when the participant hovered the mouse cursor over the asterisk and sustained as the participant moved the location of this item. Other than that, the procedure of the linguistic MA was identical to the visual MA.

Although the high-level representation is instantly accessible through direct perception in the visual modality, the representation in the linguistic modality is covert and relies on mental reconstruction of the sentence content. This introduced the possibility that the linguistic MA is cognitively more demanding than the visual MA task (i.e., reconstructing the mental image and recruiting working memory to preserve its representation). To control for the cognitive load of the overt visual and covert linguistic modality, we introduced a control condition in which the natural scene images were initially displayed as white rectangles (Supplementary Figure S1A). As was the case for captions in the linguistic MA task, in the control task the images appeared only when participants hovered the mouse cursor over the white rectangle and as they moved the item in the arena. This method allowed us to use the covert characteristic of the linguistic modality where participants are required to mentally reconstruct and maintain the representation. Other than that, the procedure was the same as in the remaining two tasks.

#### Estimating the representational dissimilarity matrices

The MA method used the weighted-averaging approach described in (Kriegeskorte & Mur, 2012) to obtain the representational dissimilarities. Following the first MA trial in which the full set of 100 stimuli was displayed, an algorithm computed Euclidean distances between every pair of stimuli ((100 items^2^ − 100)/2 = 4950 pairs) inside the MA arena and assembled them in an initial representational dissimilarity matrix (RDM) symmetric about a diagonal of zeros. Subsets of stimuli that showed the weakest weighted evidence within the RDM were then presented in subsequent MA trials. With every new trial, the representational dissimilarities in the initial RDM were iteratively adjusted by the weighted-averaging approach reducing potential placement errors. Because perceived dissimilarity between scenes is context dependent, this procedure also allowed us to account for the multidimensional nature of similarity relations. The MA trials terminated either when enough evidence was obtained for every pair of stimuli or once 45 minutes elapsed. This yielded three modality-specific RDMs per participant. Recurrent convolutional neural networks (RCNNs) trained on category- and LLM embeddings of sentence descriptions

We next constructed model-based RDMs from RCNNs to test whether artificial neural networks trained with different objectives converge on representational geometries similar to those observed in human behavior. The set of 100 natural scene images was passed through two RCNNs whose objective was to produce either a sentence caption or a category label of the image. The architecture of the network was introduced in detail in (Doerig et al., 2025). Briefly, the network was derived from vNet and was adapted to include both lateral and top-down recurrent connections. This architecture allowed us to investigate how behavioral RDMs align with recurrent activity found to correspond to representational dynamics of the visual system (Kietzmann et al., 2019). As described in (Doerig et al., 2025), both RCNNs shared identical architecture and were trained with 10 random seeds on the MS COCO dataset with the NSD stimuli excluded. The only difference between the sentence- and category-trained RCNN is in the objective of the output layer. The sentence-trained RCNN minimized the cosine distance between the predicted and the target sentence embedding obtained from MPNet. The category-trained RCNN, on the other hand, used multi-hot encoding to predict the category label. After evaluating both RCNNs on the set of 100 natural scene images, we extracted sentence embeddings and category vectors for the last layer and time step from each of 10 random seeds. We calculated correlation distance between the output vectors from each RCNN and assembled the dissimilarity estimates in model RDMs.

### Construction of baseline visual and linguistic RDMs

To provide baseline estimates of purely visual and purely linguistic representational structure, we additionally constructed model RDMs from AlexNet (Krizhevsky et al., 2017), DINOv2 (Oquab et al., 2023), and MPNet (Song, Tan, Qin, Lu, & Liu, 2020). AlexNet served as a conventional supervised visual baseline, whereas DINOv2 was included as a more recent self-supervised vision transformer with stronger performance on natural images. MPNet served as a linguistic baseline by providing sentence embeddings independent of recurrent image-to-language training. The set of 100 natural scene images was passed through AlexNet and DINOv2, and the corresponding sentence captions were embedded using MPNet. Activation vectors were extracted from each model, pairwise correlation distances were computed across all stimuli, and the resulting dissimilarities were assembled into model RDMs.

A detailed description of the NSD (http://naturalscenesdataset.org) is provided elsewhere (Allen et al., 2022). The NSD dataset contains measurements of functional magnetic resonance imaging (fMRI) responses from eight participants who each viewed 9000–10,000 distinct color natural scenes (22,000–30,000 trials) over the course of 30–40 scan sessions. Scanning was conducted at 7T using whole-brain gradient-echo EPI at 1.8-mm resolution and 1.6-second repetition time. Images were taken from the MS COCO database (Lin et al., 2014), square cropped, and presented at a size of 8.4° × 8.4°. The special set of 100 images used in our MA task was shared across subjects. Images were presented for three seconds with one-second gaps in between images. Subjects fixated centrally and performed a long-term continuous recognition task on the images. The fMRI data were pre-processed by performing one temporal interpolation (to correct for slice time differences) and one spatial interpolation (to correct for head motion). A general linear model was then used to estimate single-trial beta weights. Cortical surface reconstructions were generated using FreeSurfer, and both volume- and surface-based versions of the beta weights were created. In this paper, we used the 1.8-mm volume preparation of the NSD data and version 3 of the NSD single-trial betas (betas_fithrf_GLMdenoise_RR).

### Construction of searchlight brain RDMs

We constructed searchlight brain RDMs to characterize the local representational geometry evoked by viewing the natural scene images. The RDMs were derived from the NSD single-trial beta estimates corresponding to the Special100 image set described above (Allen et al., 2022). For each participant, activity patterns were extracted within a spherical searchlight with a radius of 6 voxels and pairwise correlation distances between activity patterns were computed to assemble searchlight brain RDMs.

### Statistical analysis

We used a cross-validated modeling approach to quantify how well behavioral similarity structure—derived from visual and linguistic MA tasks—explains representational geometry in the brain and representational structure in category- and sentence-trained RCNNs. Behavioral RDMs across all sessions were used unless stated otherwise.

The 100 stimuli were split into train and test sets at 70:30 ratio using 10-fold cross-validation. Within each fold, dissimilarities from the behavioral and observed target RDMs (brain or RCNN RDM) were split accordingly. We fit the training dissimilarities using non-negative least squares (NNLS). A major advantage of this approach is that it allows us to assign flexible weights each representing a participant's relative fit to the train subset of the observed RDM. The NNLS weights were then applied to create a behavior-predicted RDM that is the best possible representation of the observed target RDMs. This was done using the dot product between the NNLs weights and the test subset of behavioral RDMs. We then evaluated the fit between the behavior-predicted RDM and observed target RDM using Pearson correlation.

For searchlight brain RDMs, this procedure was repeated for each searchlight RDM and each NSD participant separately. Pearson correlations were averaged across folds for each NSD participant and projected from the original 1.8-mm volume to fsaverage using nsdcode (https://github.com/cvnlab/nsdcode/tree/master). Group maps were obtained by averaging Pearson correlations across NSD participants and visualized using pycortex (Gao, Huth, Lescroart, & Gallant, 2015) and nilearn (https://github.com/nilearn/nilearn). Statistical significance was assessed with one-sided *t*-test across participants and corrected for multiple comparisons at FDR *p* < 0.05.

For RCNN comparisons, Pearson correlations were averaged across the 10 random seeds for each layer and time step and tested using one-sided *t*-test with FDR correction (*p* < 0.05). Differences between sentence- and category-trained RCNNs were assessed by subtracting correlations and testing the resulting differences against zero (one-sided *t*-test; FDR *p* < 0.05).

To estimate modality-specific contributions to brain predictions, we fitted an additional joint NNLS model in which visual and linguistic behavioral RDMs were entered simultaneously to predict the searchlight brain RDMs. Predictive fits from the joint and single-predictor models were converted to *R*^2^ values by squaring the cross-validated Pearson correlations between behavior-predicted and observed brain RDMs. Unique linguistic contribution was estimated as the difference between the joint model and the visual-only model (*R*^2^_joint_ – *R*^2^_visual_), whereas unique visual contribution was estimated as the difference between the joint model and the linguistic-only model (*R*^2^_joint_ – *R*^2^_linguistic_). Statistical significance of the resulting group maps was assessed using one-sided *t*-tests across participants.

## Results

### Uncovering a shared similarity space between visual and linguistic stimuli

To investigate the overall overlap between the similarity judgments in vision and language, we used the fixed-effects representational similarity analysis approach in which we averaged the modality-specific RDMs across all participants and compared them using Spearman correlation. We found a strong relationship between the visual and linguistic RDMs (Figure 1B, fixed effects Spearman ⍴ = 0.781). A similar effect was observed when we accounted for inter-individual variability using the random-effects approach, where we computed pairwise correlations across all individuals in the visual and linguistic MAs (random effects Spearman ⍴ = 0.161, *p <* 0.0001). Importantly, we observed significant overlap between the modalities in initial experimental sessions where neither of the groups were familiar with the content of the other modality (Supplementary Figure S1B). We next compared within-modality RDM correlations in Session 1 to quantify the consistency of behavioral similarity spaces across subjects. This showed robust across-participant consistency, suggesting some commonalities in similarity judgments across both tasks. Interestingly, we observed a significantly greater agreement for the visual MA, indicating that sentence captions engaged more idiosyncratic judgments (Supplementary Figure S1C). Together, these findings demonstrate that even simple sentence descriptions preserve enough information for similarity relations to align with those derived from natural scene images, while still allowing greater individual variability than observed in the visual task.

### Visual and linguistic similarity spaces predict visually evoked brain activity in areas aligned with LLM embeddings of scene captions

**Figure 2.**
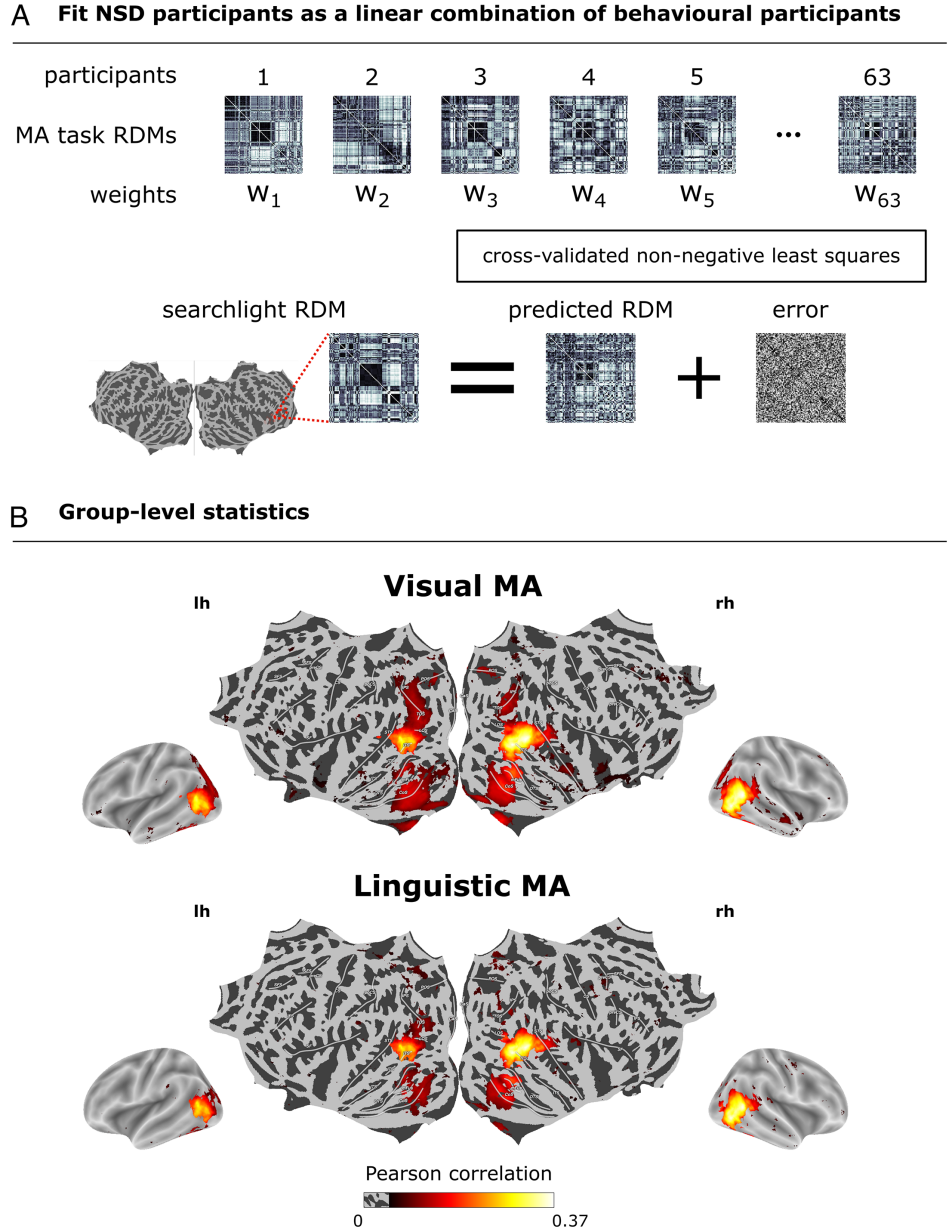
**Representational alignment of behavior-predicted and observed brain RDMs in natural scene viewing.** (**A**) Cross-validated non-negative least squares regression was used to model the brain RDMs at every searchlight location using the behavioral RDMs derived from our MA tasks. (**B**) We averaged Pearson correlations across folds and determined significance across participants using a one-sided *t*-test (FDR corrected *p* < 0.05 level). The top panel displays alignment between observed brain RDMs and behavior-predicted brain RDMs from the visual modality. The bottom panel displays alignment with behavior-predicted brain RDMs from the linguistic modality.

The results above show that similarity judgments in vision and language lead to strikingly similar representational geometries in behavior, but they leave unclear whether this reflects similar brain representations. To address this gap, we used the visual and linguistic similarity judgments to model fMRI responses to viewing the natural scene images in an independent group of eight NSD participants. Figure 2B shows that behavior-predicted RDMs, whether based on visual (Figure 2B top panel) or linguistic similarity judgments (Figure 2B bottom panel), both exhibit a similar alignment with observed brain RDMs in regions located bilaterally along the mid- and high-level visual areas. The group-averaged Pearson correlations peaked at 0.363 for the visual modality and 0.353 for the linguistic modality. Results for each NSD participant are similar to group-level results (Supplementary Figure S2). These results show that similarity judgments of images and sentences align with much the same high-level visual brain regions.

Because participants completed similarity judgments on both images and sentences, it is possible that the alignment of similarity judgments across modalities stemmed from participants’ familiarity with the stimuli. Specifically, participants may have intentionally arranged sentences in Session 3 much as they arranged images in the preceding session, potentially obscuring any differences between the modalities. To test whether the alignment between the behavior-predicted and observed brain RDMs persists even before exposure to the other modality, we performed an additional analysis in which we predicted the brain RDMs using only behavioral RDMs from Session 1. The results revealed a significant representational alignment between the Session 1 behavior-predicted RDMs and observed RDMs with correlation peaks in the occipitotemporal cortex (Supplementary Figure S1D) similar to what was found in Figure 2. These results demonstrate that it is possible to reliably model visual responses using similarity judgments of sentence captions that were made by participants who had never seen the corresponding images before.

Alternatively, the representational alignment could be driven by the NNLS fitting procedure, which may bias the predicted RDMs toward an optimally weighted combination of behavioral participants, potentially inflating prediction accuracies. To rule out this possibility, we correlated the averaged modality-specific behavioral RDMs from Session 1 with observed RDMs at every searchlight. Supplementary Figure S1E reveals significant alignment in the occipitotemporal cortex for both visual and linguistic modalities, similar to results in Figure 2. This control analysis shows that the representational alignment is not merely an artifact of NNLS fitting. Notably, this holds despite using behavioral RDMs from Session 1, eliminating the possibility that familiarity played a role. Together, these findings support the idea that the representational geometry of visually evoked responses is shared with the psychological similarity structure of natural scene images and sentence captions.

#### Joint modeling of visual and linguistic behavioral predictors

To further assess whether either modality contributed predictive information beyond the substantial shared structure observed above, we fitted an additional joint NNLS model in which visual and linguistic behavioral RDMs were entered simultaneously to predict searchlight brain RDMs using Session 1 behavioral data only. The joint model reproduced the same bilateral mid- and high-level visual pattern observed in the single-predictor analyses (max Pearson correlation = 0.34, Supplementary Figure S3A), indicating that most predictive variance was shared across modalities. We then estimated modality-specific residual contributions by comparing the joint model with each single-predictor model in *R*^2^ space. Although effect sizes were modest overall and did not survive FDR correction, thresholded maps at one-sided *p* < 0.05 showed that linguistic similarity retained broader residual predictive effects across higher-level visual cortex, with a more expansive spatial pattern in the left hemisphere (max *R*^2^ = 0.025), whereas visual similarity produced more focal residual clusters centered primarily in right ventral visual cortex (max *R*^2^ = 0.037; Supplementary Figures S3B, S3C). These results further suggest that visual and linguistic similarity judgments share a largely overlapping predictive structure while retaining limited modality-specific variance in higher-level visual regions.

### LLM-trained visual ANNs capture visual and linguistic similarity spaces

Which computational models best capture the structure of these visual and linguistic similarity spaces? To investigate this, we tested two types of visual Recurrent Convolutional Neural Networks (RCNNs) that take images as input: one was trained to predict scene category labels, while the other was trained to predict LLM embeddings of scene captions generated by MPNet (Doerig et al., 2025). This was motivated by a vast literature showing that object categories play a major role in ventral stream representations (Güçlü & van Gerven, 2015; Ishai, Ungerleider, Martin, Schouten, & Haxby, 1999; Khaligh-Razavi & Kriegeskorte, 2014; Kriegeskorte, Mur, Ruff, et al., 2008; Martin, Wiggs, Ungerleider, & Haxby, 1996; Ungerleider & Haxby, 1994). However, recent evidence suggests that training ANNs to predict LLM embeddings of scene captions from visual inputs may be even more effective in modeling brain activities during natural scene viewing (Doerig et al., 2022). Aside from the training objective, the category- and LLM-trained RCNNs were identical in their architecture, training data, and random seeds. This ensured that any differences in their fit to human similarity judgments were driven solely by the training objectives.

**Figure 3.**
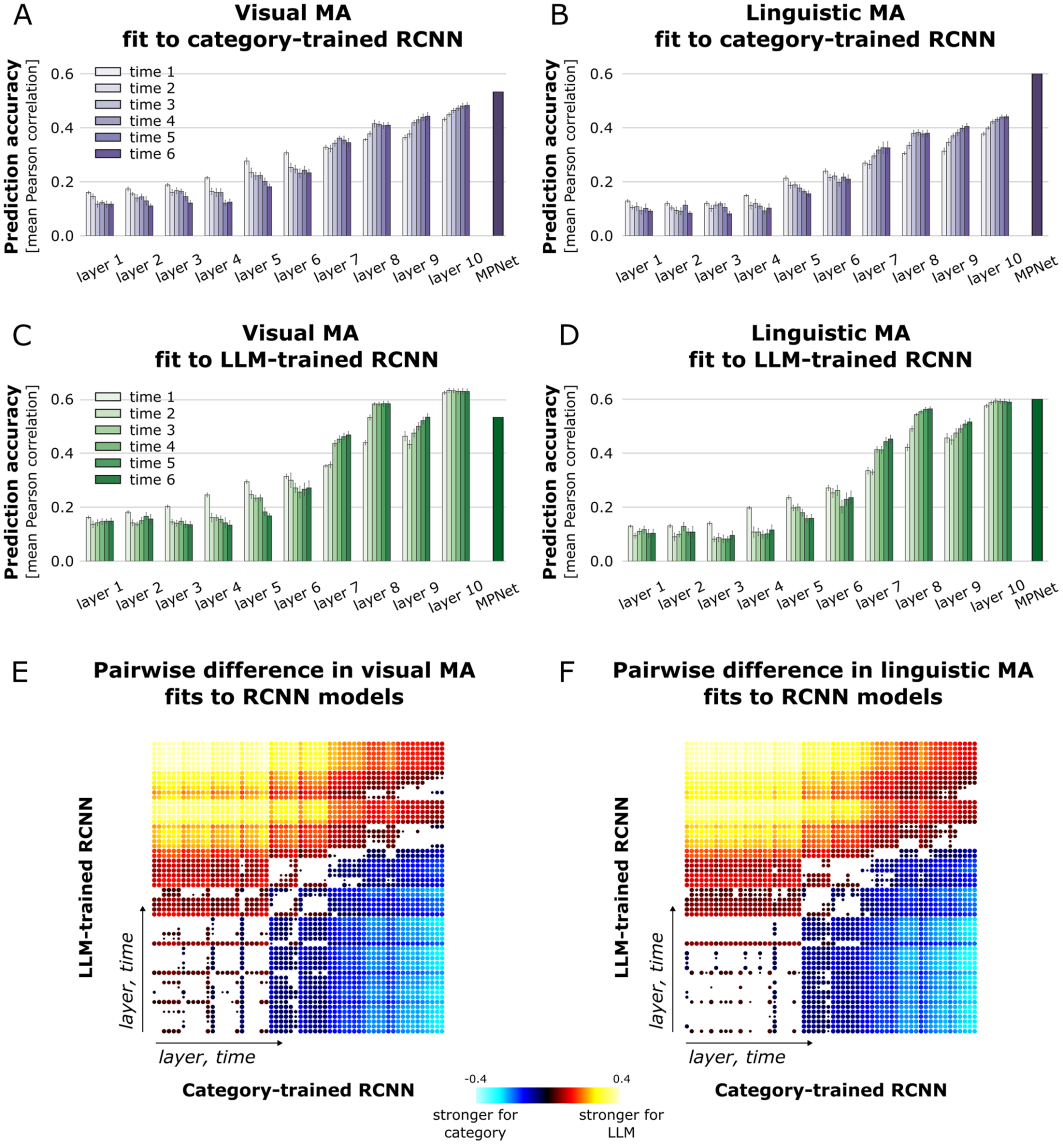
**Representational alignment between behavior-predicted RCNN RDMs and observed RCNN RDMs.** Bars indicate Pearson correlation between behavior-predicted RCNN RDMs and observed RCNN RDMs for each layer and time step, averaged across 10 models trained with different random seeds. Black bars represent Pearson correlation between the behavior-predicted MPNet RDM and observed MPNet RDM, computed from the embeddings of scene captions. Error bars show the standard error of the mean, estimated across 10 cross-validation folds for each seed. Panels (**A**) and (**B**) display prediction accuracies for category-trained RCNNs based on visual and linguistic MA, respectively. Panels (**C**) and (**D**) show prediction accuracies for LLM-trained RCNNs based on visual and linguistic MA, respectively. Pairwise difference in prediction accuracies between LLM- and category-trained RCNNs was calculated to identify a better fitting RCNN in the visual (**E**) and linguistic MA (**F**). Two-sided t-tests were performed on Pearson correlations across seeds for each layer and time step to assess significant differences between RCNN RDM fits to behavior-predicted RDMs. Only statistically significant differences are shown. Point radius reflects p-value size (*p* < 0.05 FDR corrected), and color gradients indicate the magnitude of the difference, calculated by subtracting category-trained from LLM-trained RCNN prediction accuracies. Layer and time complexity increase along both axes from the origin.

We created behavior-predicted RDMs using the cross-validated NNLS procedure described in Methods. We then compared the behavior-predicted RCNN RDMs to the observed RCNN RDMs either from the category- or LLM-trained RCNN to investigate which model, layer, and timestep best captures the representational structure of human similarity judgments. We found that the behavioral RDMs predicted the representational structure of LLM-trained RCNNs significantly better than that of category-trained RCNN; this effect was observed for both visual and linguistic modality from layer 4 onward (Figures 3E, 3F). Behavior-predicted RDMs also aligned significantly better with later time steps within each layer, regardless of the RCNN type, but the opposite trend was observed for layers 6 and lower (Figures 3A–F, see Supplementary Figures S4A–D for within-RCNN comparisons across layers and timesteps). In control analysis we found that the LLM-trained RCNN outperformed Alexnet. It also outperformed MPNet embeddings (i.e., the LLM embeddings used to train our LLM-trained RCNN) in the visual modality, although not in the linguistic modality (Supplementary Figures S4E, S4F). Together, these findings show that LLM-trained RCNNs outperform category-trained controls at predicting similarity judgments, suggesting that the multidimensional, embedding-like structure of these models better reflects how humans encode knowledge about the world.

## Discussion

Humans can effortlessly describe what they see, and “see” what they read, but establishing a shared representational format between vision and language has been a challenging task. Here, we collected similarity judgments of natural scene images and associated sentence captions to investigate whether these modalities have a shared similarity structure. The results reveal that similarity judgments of sentences closely mirror those of images, and even reliably predict brain responses to natural scene viewing in an independent group of participants. Importantly, these brain predictions emerge even when the linguistic similarity judgments were made by individuals who had not yet seen the corresponding images. These findings suggest the existence of a shared representational format that arises from the mind's ability to recognize relational structure between concepts, independent of the first-order visual experience. Furthermore, a comparison with state-of-the-art computational models reveals that behavioral similarity judgments align most closely with the rich, semantic structure of LLM-based models, offering an insight into how concept representations are formed in the human mind.

The idea of second-order isomorphism has been considered as a core organizational principle of conceptual representations (Shepard & Chipman, 1970). This idea posits that the mind's internal representations preserve the relational structure of objects observed in the environment. While this account has been applied primarily to vision–examining how similarity judgments of visual objects relate to brain activity during object viewing (Charest, Kievit, Schmitz, Deca, & Kriegeskorte, 2014; Cichy, Kriegeskorte, Jozwik, van den Bosch, & Charest, 2019; Contier, Baker, & Hebart, 2024; Mur et al., 2013), its role in other modalities has remained underexplored. We show that a similar relational structure emerges for both linguistic and visual inputs. This finding is consistent with that of (Dima et al., 2024) who showed overlap between similarity judgments of actions in videos and sentences, extending the early proposals of second-order isomorphism to language. It may be tempting to interpret the overlap in similarity judgments as evidence for amodal representations that emerge in supramodal brain regions and constitute modality-neutral conceptual information (Fairhall & Caramazza, 2013). However, our brain predictions do not immediately support this interpretation, showing that the relational structure closely resembles how visual experience is encoded in the visual system.

Importantly, we found that not only visual, but also linguistic similarity judgments predicted brain responses to natural scene images across mid- and high-level visual regions. This effect persisted even when participants had not seen the images before judging the similarity of captions. An additional variance-partitioning analysis yielded modest modality-specific effects, with residual linguistic contributions appearing more broadly distributed across bilateral high-level visual regions, including a greater extent in the left hemisphere. This descriptive pattern is consistent with recent evidence suggesting that linguistic feedback may contribute to visual representations in the ventral occipitotemporal cortex (Chen et al., 2025; B. Liu et al., 2025). We further observed that the single-predictor correlation maps appeared somewhat stronger in the right hemisphere and in lateral relative to ventral visual cortex at the group level. The apparent bias may reflect the predominance of high-level scene and spatial processing, which has often been associated with relatively stronger right-hemisphere engagement (Roser, Fiser, Aslin, & Gazzaniga, 2011). Residual visual contributions in the variance-partitioning analysis appeared relatively focal and centered primarily in right ventral visual cortex, suggesting that part of the apparent rightward bias may reflect modest modality-specific visual variance. However, because hemispheric lateralization was not a primary focus of the present study, and modality-specific effects remained small, these spatial differences should be interpreted cautiously and warrant more systematic investigation in future work.

We speculate that the alignment of linguistic similarity judgments and responses to natural scene images suggests that, while text and natural scene images have different visual features, they both ultimately project to the same abstract, high-level representational space. This interpretation is consistent with recent work suggesting that high-level visual cortex uses a highly abstract representational format well modeled by complex features such as object co-occurrences (Bonner & Epstein, 2021) or LLM embeddings (Doerig et al., 2025). At the same time, the unique variance analysis indicates that this convergence is not complete: visual and linguistic similarity each retained modest modality-specific predictive structure, suggesting that they also preserve partially distinct aspects of scene representation. One possibility is that the visual system translates sensory input into stable relational format that can also be reactivated through language, while preserving modality-dependent traces tied to perceptual versus linguistic access to the same world structure. Identifying which semantic dimensions contribute to this shared versus unique organization remains an important direction for future work.

Further analyses revealed that the representational structure encoded in the human mind also closely resembles the way several LLM-based computational models encode information. More specifically, we found that behavioral similarity judgments better predicted the feature space of LLM-trained RCNNs and LLM embeddings than that of category-trained RCNNs and visual baseline models. The stronger alignment between behavioral similarity judgments and LLM-based models suggests that human conceptual structure may be better captured by high-dimensional, relational embedding spaces than by models trained to assign discrete category labels. Category-trained RCNNs collapse natural scenes into fixed, orthogonal labels, potentially discarding the rich relational structure that similarity judgments rely upon. Importantly, the category- and LLM-trained RCNNs share identical architecture, training data, and random seeds, such that the observed advantage of LLM-trained models cannot be explained by differences in architecture, initialization or dataset size, leaving the training objective as the primary distinguishing factor (Doerig et al., 2025).

A further consideration is whether the comparatively weaker alignment of category-trained RCNNs reflects limitations of training models to predict discrete category labels, rather than fundamental differences in representational format. Although the category- and LLM-trained RCNNs share identical structure, it remains possible that approximating human visual cortex requires substantially greater architectural complexity (Mahner, Muttenthaler, Güçlü, & Hebart, 2025). It is important to note, however, that LLM-trained RCNN outperform not only the category-trained RCNN, but also the ImageNet-trained AlexNet with greater training data and higher late-layer dimensionality and the contemporary vision transformer DINOv2. This suggests that model capacity or dataset size alone cannot explain the observed differences. Our findings are consistent with the view that training objectives capturing rich statistical co-occurrence structure through linguistic feedback—rather than data complexity alone—may be critical for approximating the relational geometry reflected in human similarity judgments and neural representations (Chen et al., 2025; B. Liu et al., 2025; Wang et al., 2023). Future work comparing more recent vision architectures, including transformer-based models or multimodal foundation models, will be important for clarifying the extent to which architectural complexity versus linguistic training objective drives alignment with human conceptual structure.

We further found that visual similarity judgments predicted LLM-trained RCNNs significantly better than purely linguistic MPNet embeddings, whereas linguistic similarity judgments predicted both models equally well. This asymmetry suggests that the shared similarity structure may retain traces of modality-specific information. Visual judgments likely preserve perceptual detail that is not fully captured by sentence captions, which may explain their stronger alignment with LLM-trained RCNN, where images are processed before being projected into the embedding space and may therefore retain traces of visual structure (Collell & Moens, 2016; Rubinstein, Levi, Schwartz, & Rappoport, 2015; Zhuang, Fedorenko, & Andreas, 2024). In contrast, linguistic judgments may rely more strongly on abstract semantic structure, allowing them to align equally well with both MPNet embeddings and the LLM-trained RCNN, both of which encode statistical regularities in language. An open question is where the broader residual linguistic effects in the variance-partitioning analysis may partly reflect representational geometry that is not fully reducible to visual similarity. Future work should directly compare modality-specific representational dimensions in vision and language against the universal structure shared between them to clarify how behaviorally relevant knowledge emerges across systems.

Overall, behavior-predicted RDMs showed increasing alignment with later RCNN layers, while alignment was weaker in the earliest layers. This pattern is consistent with the hierarchical organization of the models: early layers primarily encode low-level perceptual features whereas later layers capture increasingly abstract and semantically structured representations. Unless participants are specifically instructed to judge similarity on the basis of low-level properties, behavioral similarity judgments are unlikely to reflect early perceptual feature maps directly but instead operate over higher-order conceptual structure (Cichy et al., 2019). The stronger alignment with later layers therefore suggests that human similarity judgments rely on abstract relational representations rather than low-level visual properties. Within layers, we also observed that behavioral alignment tended to decrease across recurrent steps in early layers but increase in later layers. One possible interpretation is that recurrent processing in early layers progressively refines low-level feature structure in a way that becomes less behaviorally relevant, whereas in later layers recurrence may further stabilize abstract representational structure that more closely matches human similarity judgments.

In this study, we do not test how behavioral representational geometries align with neural activity during sentence reading. This leaves open the question of whether reading the captions would recruit the same neural populations that we identified in fMRI evoked by viewing the natural scene images. Further research will therefore require new MRI data collection. However, this matter has been partially addressed in recent work, in which participants either viewed natural scene images or read sentence captions during fMRI data collection (Nikolaus et al., 2024). This work has found that decoders trained on activity in high-level visual areas perform well both for decoding images and captions, indicating that patterns of brain activity elicited during sentence reading overlap with patterns elicited during scene viewing. Similar effects were observed during movie watching and story listening ((Popham et al., 2021; Tang et al., 2023). These findings suggest that future fMRI studies of sentence reading may uncover a similar predictive relationship between similarity judgments and activity patterns in mid- and high-level visual regions, as observed in the current study. Future research should address whether this relational similarity structure constitutes a universal principle of conceptual representations. If so, it may extend beyond vision to other domains such as sound or motion perception (Barsalou, 2016). Indeed, prior work has demonstrated that similarity among action-related concepts–whether conveyed through language or visual stimuli–can predict neural patterns in motor and premotor regions (Hauk, Johnsrude, & Pulvermüller, 2004; Oosterhof, Wiggett, Diedrichsen, Tipper, & Downing, 2010).

A potential concern is that the observed convergence between visual and linguistic similarity structures may partially reflect task-related factors rather than a fully modality-independent representational format. Although the linguistic MA task required participants to arrange sentence captions, it is possible that participants engaged visual imagery when interpreting the scenes described in language. If so, linguistic similarity judgments may incorporate elements of visually grounded representation, thereby increasing their alignment with visually derived similarity structure. However, several aspects of the results suggest that this convergence cannot be reduced entirely to shared visual imagery. First, MPNet, which does not have access to perceptual input, showed similar alignment patterns, indicating that abstract statistical structure in language alone may approximate aspects of the observed geometry. Second, the differential fits of visual and linguistic behavioral RDMs to computational models suggest that each modality retained partially non-overlapping information. This interpretation was further supported by the joint NNLS analysis, which revealed modest but spatially dissociable unique contributions of visual and linguistic similarity to brain predictions. Together, these findings suggest that the observed alignment reflects a mixture of shared conceptual structure and modality-specific contributions, rather than a purely task-driven convergence. Future work should identify which semantic dimensions account for this shared geometry and determine the extent to which cross-modal alignment reflects intrinsic conceptual structure versus task-induced cross-modal grounding.

## Conclusions

We draw several core observations from our results. First, these results support the idea that behavioral judgments of similarity in vision and language converge in the way they encode the relational structure of the physical world, independent of the first-order visual similarity. This relational structure closely mirrors how the visual system encodes sensory inputs in high-level visual areas. One exciting possibility is that this structure may reflect a general organizational principle for conceptual representations across modality-specific brain regions. Furthermore, we found that LLM-based models best accounted for the behavioral similarity structure, underscoring the richness and granularity of information they encode. Together, these findings provide a neural foundation for how concepts are encoded in the brain, revealing a universal representational structure that links brain, behavior, and computational models.

## Supplementary Material

Supplement 1

Supplement 2

Supplement 3

Supplement 4
