# Supplementary material for: Representations in vision and language converge in a shared, multidimensional space of perceived similarities
Source: J Vis. 2026 May 20;26(5):7. doi: 10.1167/jov.26.5.7 (PMC13206752; doi:10.1167/jov.26.5.7)
Supplement: Supplement 1 [file jovi-26-5-7_s001.pdf]

# Supplementary Figure 1

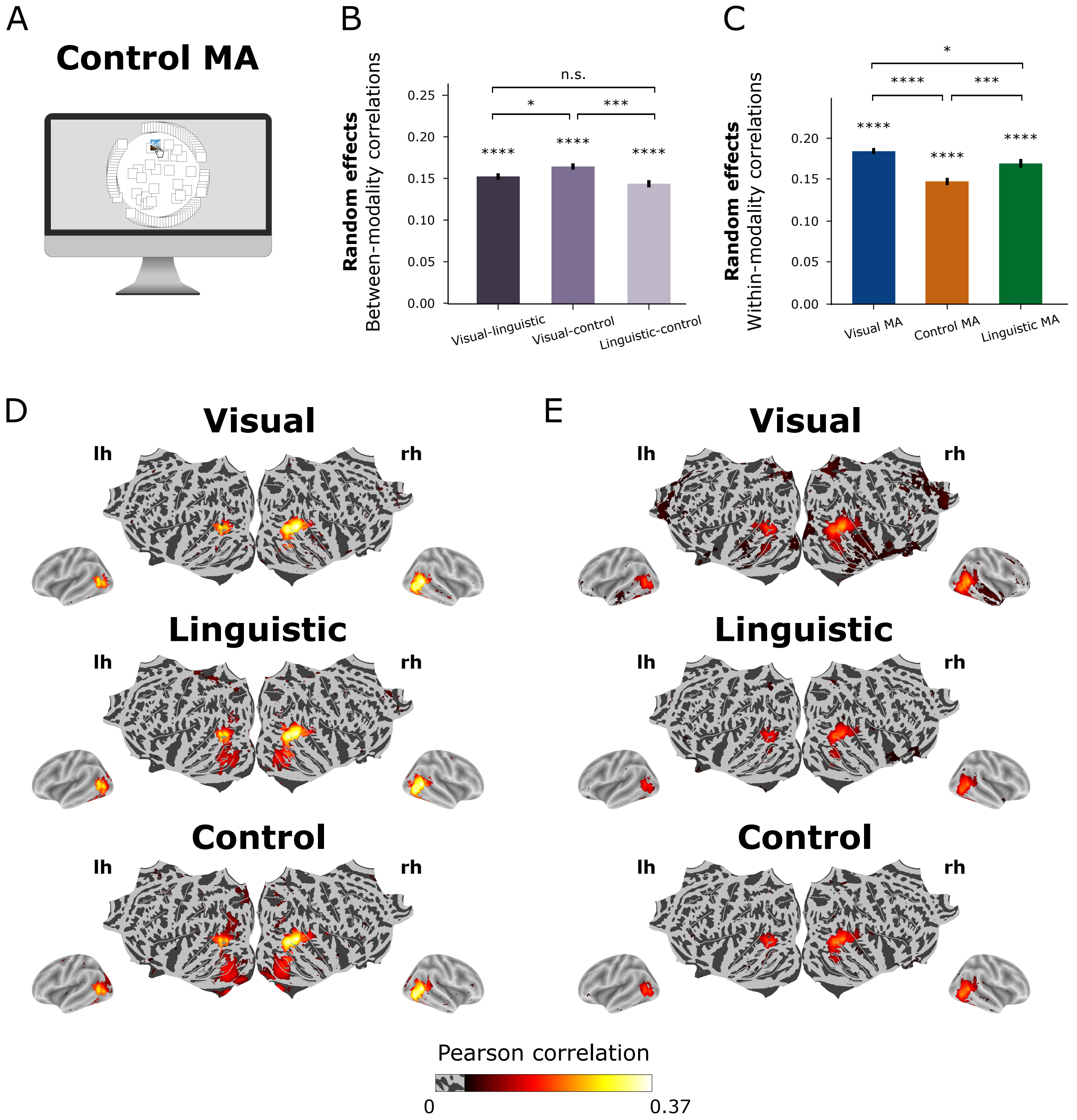

Supplementary Figure 1. (A) Experimental design of the control MA task. Participants were presented with the same set of 100 natural scene images as used in the visual MA, only these images were displayed as white rectangles. The images appeared one at a time as the participant dragged or hovered over them with the mouse cursor. The same procedure was followed as in the other MA tasks. (B) Alignment in similarity judgements in Session 1. The bars display average between-modality correlations across all pairwise RDM combinations (i.e., random effects analysis) for RDMs from Session 1 only. This enabled us to investigate the representational overlap before participants were familiar with the content of the other modalities than they started with. Independent samples t-test revealed that the control MA is significantly better aligned with the visual MA compared to the linguistic MA. No difference was found, whether the linguistic MA was correlated with the visual or control MA. (C) Within-modality reliability of behavioural similarity judgements. Bars display the mean across-participant correlation of RDMs within each modality on Session 1 similarity judgements. Visual MA exhibited the highest inter-participant consistency, followed by linguistic MA, with the control MA showing the lowest consistency (pairwise comparisons, all $p < 0.05$). (D) Alignment between behaviour-predicted RDMs from Session 1 and observed brain RDMs. The figure shows group-averaged Pearson correlations between the behaviour-predicted and observed brain RDMs ($p < 0.05$, FDR-corrected, one-sided test). The surface maps reveal a significant representational overlap between the RDMs spanning the occipitotemporal cortex similar to what was shown in Figure 2. The Pearson correlations peak at 0.347, 0.338, and 0.346 for visual, linguistic, and control MA respectively. (D) Alignment between behavioural similarity judgements from Session 1 and visually evoked brain responses. The averaged modality-specific behavioural RDMs from Session 1 were correlated with brain RDMs at every searchlight for each NSD participant. The surface maps show averaged Pearson correlations across the NSD participants ($p < 0.05$, FDR-corrected, one-sided test). The correlations peaked at 0.239, 0.204, and 0.220 for visual, linguistic, and control MA respectively. The stars denote p-values with: *$p < .05$, ** $p < .01$, *** $p < .001$, **** $p < .0001$.
