# Supplementary material for: Representations in vision and language converge in a shared, multidimensional space of perceived similarities
Source: J Vis. 2026 May 20;26(5):7. doi: 10.1167/jov.26.5.7 (PMC13206752; doi:10.1167/jov.26.5.7)
Supplement: Supplement 2 [file jovi-26-5-7_s002.pdf]

Supplementary Figure 2

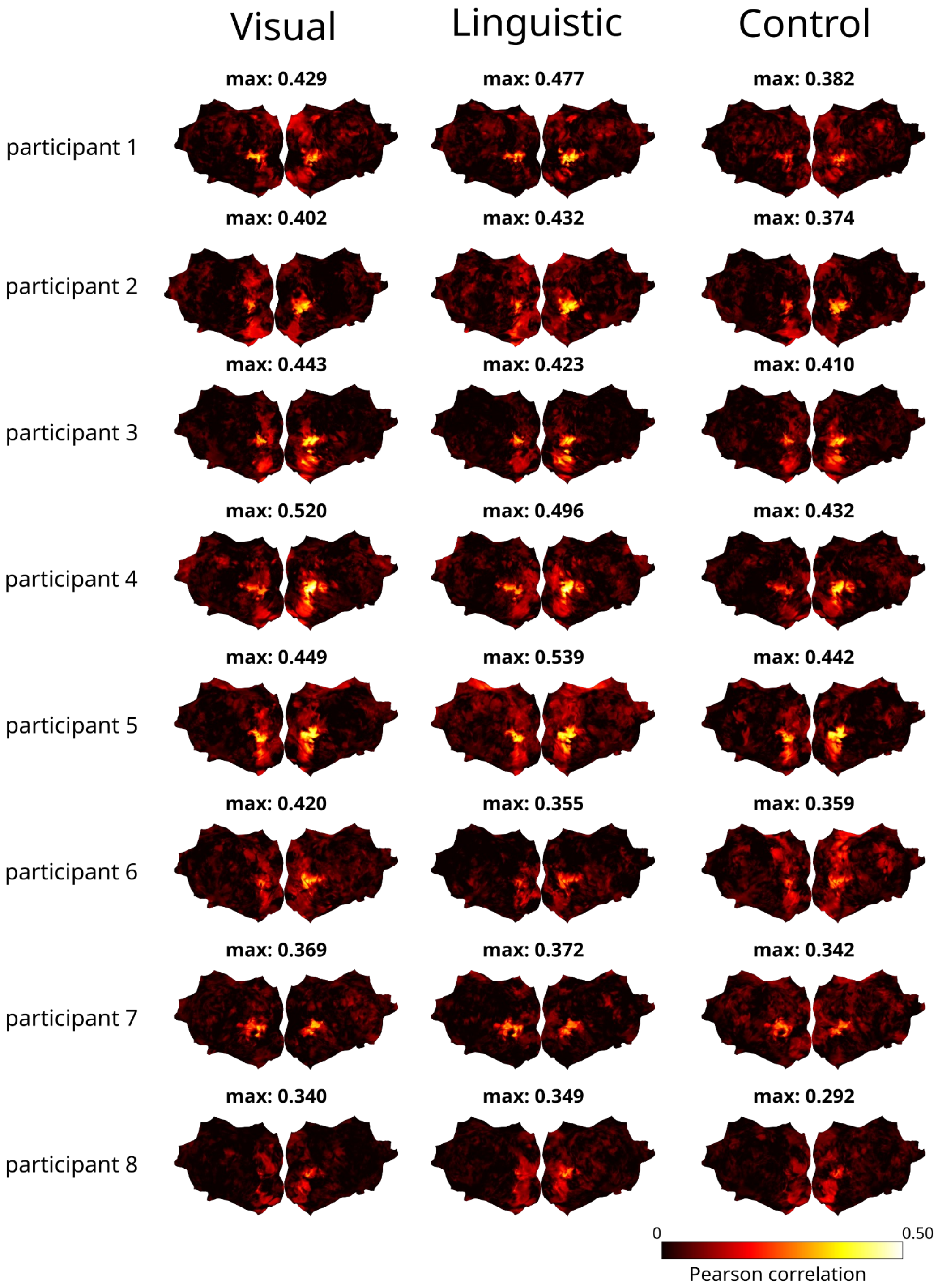

Supplementary Figure 2. Representational alignment between the behaviour-predicted and observed brain RDMs across all sessions at the participant level. For each NSD participant, Pearson correlations between the behaviour-predicted and observed brain RDMs were averaged across 10 cross-validation folds. Representational alignment is reported for all three task modalities. Similar to group-level comparisons, the surface maps reveal strong and relatively stable correlations across all NSD participants and tasks bilaterally along the occipitotemporal cortex.
