# Supplementary material for: Representations in vision and language converge in a shared, multidimensional space of perceived similarities
Source: J Vis. 2026 May 20;26(5):7. doi: 10.1167/jov.26.5.7 (PMC13206752; doi:10.1167/jov.26.5.7)
Supplement: Supplement 3 [file jovi-26-5-7_s003.pdf]

Supplementary Figure 3

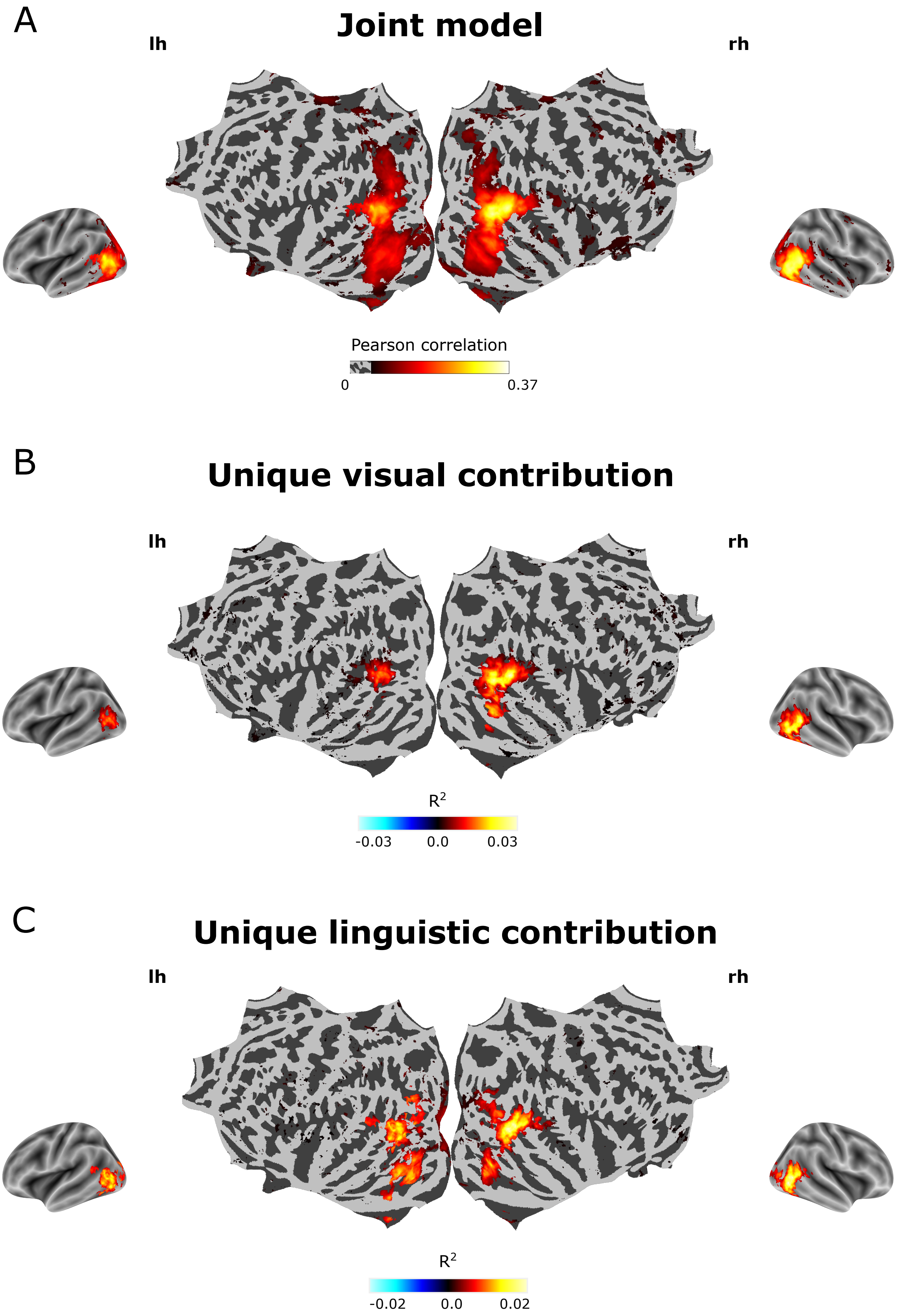

Supplementary Figure 3. Joint and modality-specific contributions of behavioural similarity judgements to brain prediction in Session 1. To assess modality-specific contributions while minimising potential effects of cross-modal familiarity, we examined differences between the joint model and single-predictor NNLS models using Session 1 behavioural RDMs only, before participants had completed the MA task in the alternate modality. (A) Predictive performance of the joint NNLS model including both visual and linguistic behavioural RDMs ($p < 0.05$, FDR-corrected, one-sided test). (B) Unique visual contribution estimated as the difference in prediction performance between the joint model and the linguistic-only model. (C) Unique linguistic contribution estimated as the difference in prediction performance between the joint model and the visual-only model ($p < 0.05$, uncorrected, one-sided test). Both modalities contributed modest modality-specific variance, with linguistic similarity showing broader unique effects across bilateral high-level visual cortex.
