# Supplementary material for: Representations in vision and language converge in a shared, multidimensional space of perceived similarities
Source: J Vis. 2026 May 20;26(5):7. doi: 10.1167/jov.26.5.7 (PMC13206752; doi:10.1167/jov.26.5.7)
Supplement: Supplement 4 [file jovi-26-5-7_s004.pdf]

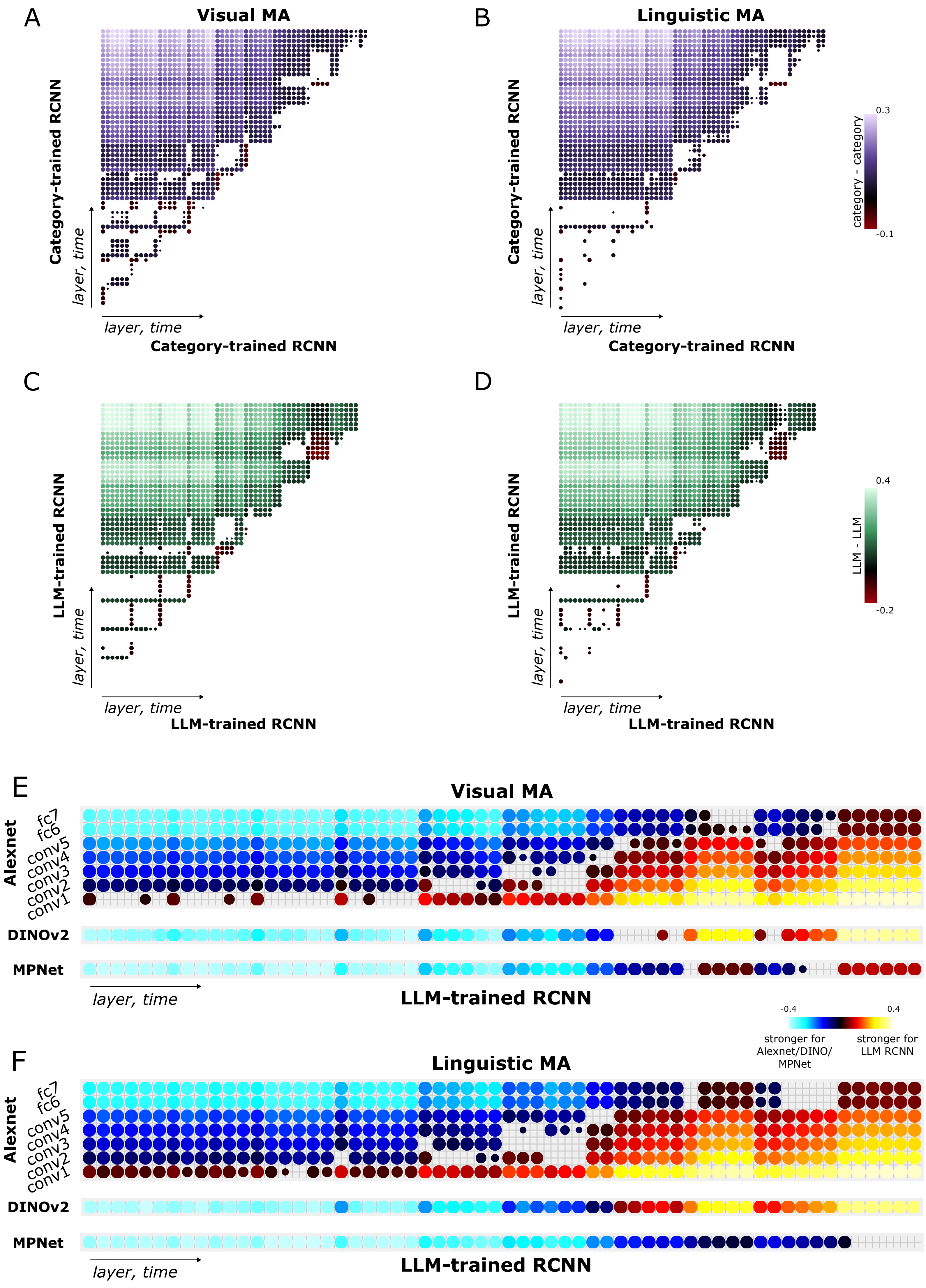

A
Visual MA
Category-trained RCNN
layer, time
layer, time
Category-trained RCNN
B
Linguistic MA
Category-trained RCNN
layer, time
layer, time
Category-trained RCNN
0.3
category - category
-0.1
C
LLM-trained RCNN
layer, time
layer, time
LLM-trained RCNN
D
LLM-trained RCNN
layer, time
layer, time
LLM-trained RCNN
0.4
LLM - LLM
-0.2
E
Visual MA
Alexnet
fc7
fc6
conv5
conv4
conv3
conv2
conv1
DINOv2
MPNet
layer, time
LLM-trained RCNN
-0.4
0.4
stronger for Alexnet/DINO/ MPNet
stronger for LLM RCNN
F
Linguistic MA
Alexnet
fc7
fc6
conv5
conv4
conv3
conv2
conv1
DINOv2
MPNet
layer, time
LLM-trained RCNN

Supplementary figure 4. (A-D) Pairwise differences in behaviour-predicted RCNN RDM fits to observed RCNN RDMs across layers and time steps. Differences in prediction accuracy were obtained by subtracting Pearson correlations for all pairwise combinations of layers and time steps within each RCNN model type. The colour gradient indicates the magnitude of the difference, and point radius denotes the p-value size ($p < 0.05$, FDR-corrected; one-sided t-test across seeds). Panels A and B depict comparisons between category-trained RCNN RDMs and behaviour predicted RDMs derived from visual and linguistic MA respectively. Panels C and D depict the same for LLM-trained RCNNs. (E-F) Pairwise differences in behaviour-predicted RCNN RDM fits to observed RCNN and baseline model RDMs. The image set was additionally processed AlexNet and DINOv2 as visual baseline models, and sentence captions were processed through MPNet sentence encoder as a linguistic baseline model. Behaviour-predicted RDMs were compared to observed RDMs from these baseline models to assess alignment. Panel E shows pairwise differences in Pearson correlations between the LLM-trained RCNN and baseline model RDMs with behaviour-predicted RDMs in the visual MA. Panel F shows the same comparison between LLM-trained RCNN and MPNet RDMs for the linguistic MA. For both panels, Pearson correlations across RCNN seeds were compared against correlations from baseline models that were averaged across folds using two-sided, one-sample t-test ($p < 0.05$, FDR-corrected). Only significant differences are shown. The colour gradient reflects the magnitude of the difference, computed as mean prediction accuracy from the baseline model subtracted from the mean accuracy of the LLM-trained RCNN. Layer and time-step complexity increase from the origin of the x-axis for all panels.
